# The indirect role of working memory in the association between perspective-taking skills and social problem-solving skills in preschool children: an age-sensitive moderated mediation in a Turkish sample

**DOI:** 10.3389/fpsyg.2026.1880860

**Published:** 2026-09-23

**Authors:** Muhammed Fatih Küçükkara

**Affiliations:** Early Childhood Education Department, Faculty of Education, Bolu Abant İzzet Baysal University, Bolu, Türkiye

**Keywords:** early childhood, executive function, moderated mediation, perspective taking, social problem solving, working memory

## Abstract

**Background:**

Perspective taking, working memory and social problem solving are central to early childhood social and cognitive development, yet they have rarely been examined jointly within a single model.

**Aim:**

The present study examined whether the association between three perspective-taking sub-dimensions (cognitive, perceptual, affective) and social problem solving in Turkish preschoolers was indirect, operating through working memory, and whether this indirect association varied with age.

**Method:**

A sample of 457 Turkish preschoolers aged 48–72 months (50.3% girls) was recruited from independent preschools and kindergarten classrooms affiliated with primary schools in Bolu. Children completed the Perspective-Taking Test for Children and the Wally Social Problem-Solving Test, and mothers completed the working-memory subscale of the Self-Regulation Skills Scale. Three separate moderated mediation models, one for each perspective-taking sub-dimension, were estimated using PROCESS Model 59 with age group as the moderator of all three paths and 5,000 bootstrap resamples.

**Results:**

Indices of moderated mediation were significant in all three models (cognitive 0.60, perceptual 0.52, affective 0.15) and indirect effects through working memory strengthened systematically with age.

**Conclusion:**

Perspective taking was indirectly associated with social problem solving through working memory, and this indirect association was stronger in older preschoolers. Because the design is cross-sectional, these patterns are associational and require longitudinal confirmation. The findings are consistent with curricular approaches that address cognitive and social–emotional components together.

## 1 Introduction

The capacity to resolve interpersonal challenges in early childhood is a foundational determinant of later peer relationships, school adjustment, and behavioral outcomes (Barnes et al., 2018; Ladd et al., 2006; Rubin and Rose-Krasnor, 1992). Yet preschool social problem-solving (SPS) interventions continue to yield heterogeneous and often modest effects. A systematic review of classroom-wide social–emotional learning programs found that only 10.5% meet the criteria for well-established interventions (Ştefan et al., 2022), and recent evidence indicates that children with lower working memory may show adverse outcomes when programs ignore individual cognitive profiles (Hatakeyama et al., 2024). This pattern reflects a deeper limitation in current practice. Educators and program designers lack a clear account of which cognitive capacities are most strongly implicated in SPS development, and they therefore cannot target with precision the components most likely to yield gains.

Two candidate contributors, namely perspective taking and working memory, have each received substantial developmental attention but have rarely been integrated within a single analytic framework. Most studies examine pairwise relationships, treat perspective taking as unidimensional, and rely on samples from Western contexts (Hughes et al., 2018; Imuta et al., 2016; Nejati et al., 2024). The evidence base therefore cannot specify how these capacities are jointly related to competent SPS, whether their joint operation shifts across the rapidly changing window from 48 to 72 months, or how these patterns unfold in non-Western contexts. To address these gaps, the present study pursues two complementary goals in a sample of 457 Turkish preschoolers aged 48 to 72 months. The first goal is to replicate established associations between perspective taking and SPS, and between working memory and SPS, within a non-Western cultural context. The second is to extend this literature by testing whether the association between three perspective-taking sub-dimensions and SPS is indirect, operating through working memory, and whether age moderates this indirect association.

### 1.1 Social problem solving in early childhood

Social problem solving is a multidimensional construct encompassing the cognitive, emotional, and behavioral processes through which individuals address interpersonal challenges (D'Zurilla and Goldfried, 1971; Spivack and Shure, 1974). Goldfried and Davison (1976) conceptualized it as a systematic process involving problem definition, solution generation, decision-making, and implementation. The social information processing (SIP) model (Crick and Dodge, 1994; Dodge, 1986) delineates how children encode peer cues, attribute intent, and select responses, while the integrated emotion-cognition model (Lemerise and Arsenio, 2000) emphasizes that effective problem-solving emerges from the interplay between emotional understanding and cognitive capability. Between ages four and five, children diversify problem-solving strategies beyond imitation toward flexible, negotiated responses (Spivack and Shure, 1974), and by age six, planning and anticipatory thinking refine these abilities (Eisenberg et al., 2015). Higher SPS predicts peer acceptance, school adjustment, and fewer externalizing behaviors (Nakamichi et al., 2021), whereas poor SPS predicts peer rejection extending into adolescence (Rubin and Rose-Krasnor, 1992).

### 1.2 Perspective taking and social problem solving

Perspective-taking is the capacity to apprehend others’ thoughts, feelings, and viewpoints (Healey and Grossman, 2018). Three components are commonly distinguished. The cognitive dimension involves inferences about others’ beliefs and intentions, the perceptual dimension concerns the adoption of a spatial or visual vantage point, and the affective dimension reflects the recognition and sharing of emotions (Flavell, 2004; Frick et al., 2014). Between three and six years, children begin to perform consistently on false-belief tasks (Wellman and Liu, 2004; Wellman, 2018), visual perspective-taking accuracy improves from age five (Frick et al., 2014), and affective perspective taking matures alongside emotion recognition (Decety and Holvoet, 2021). Core Theory of Mind components are typically consolidated by age four to five (Rakoczy, 2022), with development shaped by family environment, language, and cognitive flexibility (Devine and Hughes, 2014, 2018; Ertuğrul-Yaşar, 2024).

The theoretical link between perspective taking and SPS is grounded in the SIP architecture. Competent problem-solving depends on accurate encoding and interpretation of social cues, and these processes are enhanced by considering others’ viewpoints (Dodge, 1986). Recognizing that a peer may hold a different belief, intention, or emotional response enriches solution generation. Aras and Aslan (2018) found that the “I Can Problem Solve” program improved perspective-taking, and Viana et al. (2023) demonstrated that Theory of Mind predicted cooperative problem-solving quality in dyadic contexts. Evidence on whether social–emotional learning programs strengthen perspective-taking is mixed (Caldwell et al., 2026; Mages, 2018). This variation may reflect the sub-dimensional heterogeneity rarely modeled in this literature.

### 1.3 Working memory and social problem solving

Working memory is a limited-capacity system enabling temporary storage and manipulation of information (Baddeley, 2003). Baddeley’s multi-component model includes the phonological loop, visuospatial sketchpad, and episodic buffer coordinated by the central executive (Camos and Barrouillet, 2018; Wingfield, 2016). An alternative formulation treats working memory as the focally activated subset of long-term memory (Cowan, 2017). Preschool marks a phase of marked expansion, with gains between four and 5 years that parallel prefrontal maturation (Ahmed et al., 2022; Moriguchi and Hiraki, 2013; Tsujimoto, 2008). Higher capacity is associated with stronger attentional regulation, language, and problem-solving (Garon et al., 2008), and its influence extends to social–emotional adaptation (Diamond and Ling, 2020; He et al., 2019).

Within the SPS framework, working memory serves as the cognitive workspace in which children hold problem information, generate and evaluate solutions, and execute responses. It is the strongest executive predictor of competent responses to peer conflict in preschoolers (Caporaso et al., 2019). Working memory also maintains task requirements that allow evaluation against social norms (Schäfer et al., 2024) and carries indirect relationships between other cognitive capacities and social functioning (Bullard et al., 2024). In Türkiye, Aydoğan and Özyürek (2020) found significant correlations between memory capacity and problem-solving sophistication in preschoolers. Working memory has also been theorized as a linking capacity between perspective taking and broader social outcomes. Adopting another’s viewpoint requires simultaneously suspending one’s own and constructing a representation of the other’s (Carruthers, 2013; Devine and Hughes, 2014; Grosse Wiesmann et al., 2020; Pesch et al., 2020). Overlapping prefrontal and temporoparietal networks support both capacities (Saxe and Powell, 2006; Schurz et al., 2014), and behavioral evidence links working memory growth to Theory of Mind development (Ertuğrul-Yaşar, 2024; He et al., 2019; Shimizu, 2023).

### 1.4 The moderating role of age

In the present study, age functions as a contextual moderator rather than as a simple covariate (Hayes, 2022). The period from 48 to 72 months encompasses substantial changes in working-memory capacity (Ahmed et al., 2022; Camos and Barrouillet, 2018), perspective-taking sophistication (Frick et al., 2014; Wellman and Liu, 2004), and SPS fluency (Barnes et al., 2018; Rubin and Rose-Krasnor, 1992). At the younger end (48–60 months), still-developing working memory constrains children’s capacity to coordinate perspective-taking with problem solving (Garon et al., 2008), so the direct pathway from perspective taking to SPS may be relatively strong. As children approach 60–72 months, expanding capacity may support a shift toward indirect pathways. Perspective-taking information held in working memory would then be increasingly available when solutions are generated, rendering the indirect pathway more pronounced. This proposition aligns with the developmental cascades framework (Masten and Cicchetti, 2010), in which foundational skills scaffold more complex systems. It is also consistent with cross-cultural evidence that the relationship between executive function and Theory of Mind varies meaningfully by age and context (Nejati et al., 2024).

### 1.5 Cultural context of the present study

Across cultural contexts, the pathways linking executive function to social cognition vary meaningfully (Hughes et al., 2018; Nejati et al., 2024). Contemporary Türkiye occupies a distinctive position in this regard. The socialization goals of urban Turkish families reflect a synthesis of autonomy and relatedness orientations, in which independent coping is endorsed alongside traditional emphases on interpersonal harmony (Hofstede et al., 2010; Kağıtçıbaşı, 2017). Turkish preschoolers are thus socialized to navigate peer conflicts in ways that preserve relational harmony while exercising autonomous problem-solving. This profile cannot be assumed to map onto findings from predominantly Western samples.

Structural features of Turkish early childhood education further differentiate the context. Turkish preschools have historically followed a more didactic curriculum prioritizing motor and cognitive outcomes over socioemotional competence, and recent observational evidence indicates that teachers seldom encourage discussion or problem-solving during peer conflicts (Bugan-Kisir and Corapci, 2026). The Türkiye Yüzyılı Maarif Modeli [Ministry of National Education (MoNE), 2024b] has elevated social–emotional competence to a core early childhood priority. Yet the evidence base regarding the cognitive mechanisms underpinning this competence in Turkish samples remains thin. Turkish studies have examined a range of pairwise associations, including ToM and social competence (Etel and Yagmurlu, 2015), self-regulation and social cognition (Korucu et al., 2017), parenting and SPS (Ayık et al., 2025), age- and status-related SPS variation (Dereli-İman, 2014; Gülay Ogelman et al., 2023), and emotion understanding and peer play in relation to SPS (Bugan-Kisir and Corapci, 2026). However, no study has examined three-dimensional perspective taking, working memory, and SPS within a unified moderated-mediation framework. The present study is therefore the first to specify the three perspective-taking sub-dimensions separately, to position working memory as the intervening variable linking them to SPS, and to treat age as a condition of that indirect pathway rather than as a covariate.

### 1.6 The present study

Two complementary goals were pursued using PROCESS Model 59 (Hayes, 2022) in 457 Turkish preschoolers aged 48–72 months. The first aim, drawing on the SIP framework (Crick and Dodge, 1994; Lemerise and Arsenio, 2000) and developmental cascades theory (Masten and Cicchetti, 2010), was to replicate established direct associations between perspective taking and SPS within a non-Western cultural context. The second aim was to extend this literature by testing whether the association between the three perspective-taking sub-dimensions and SPS is indirect, operating through working memory, with age as a moderator. It was hypothesized that each perspective-taking sub-dimension (cognitive, perceptual, affective) would be associated with SPS both directly and indirectly through working memory, and that age would moderate the strength of the indirect pathway, which was expected to strengthen across the window from 48 to 72 months. Testing this integrated model makes it possible to establish whether the association between perspective taking and SPS is statistically accounted for by working memory, and to locate the developmental window in which that indirect association is most pronounced. These findings are relevant to curriculum design that addresses perspective taking and working memory together within the Türkiye Yüzyılı Maarif Modeli framework.

The following hypotheses guided the present inquiry:

H1. Each perspective-taking sub-dimension (cognitive, perceptual, affective) is positively associated with social problem solving in Turkish preschoolers.

H2. Each perspective-taking sub-dimension is positively associated with working memory, and working memory is positively associated with social problem solving.

H3. The association between each perspective-taking sub-dimension and social problem solving is indirect, operating through working memory. The indirect effect (*a* × *b*) is expected to be significantly different from zero in all three models.

H4. Age moderates the first-stage pathway from perspective taking to working memory. The strength of this pathway increases across the age range from 48 to 72 months.

H5. Age moderates the second-stage pathway from working memory to social problem solving. The strength of this pathway increases across the age range from 48 to 72 months.

H6. The indirect effect of perspective taking on social problem solving through working memory is stronger among older preschoolers. This pattern is indexed by a significant index of moderated mediation for each perspective-taking sub-dimension.

H7 (exploratory). Whether age moderates the direct effect of perspective taking on social problem solving was examined on an exploratory basis. Given the absence of a strong theoretical basis for predicting either stability or change in this pathway, no directional prediction was made.

## 2 Method

### 2.1 Design

The study employed a cross-sectional correlational design to examine the determinants of social problem-solving in Turkish preschoolers. Specifically, the indirect role of working memory in the relationship between the three perspective-taking sub-dimensions (cognitive, perceptual, affective) and social problem-solving, and the moderating role of age in this indirect pathway, were examined using PROCESS macro Model 59 (Hayes, 2022). Social problem-solving served as the primary outcome variable (Y), with perspective-taking specified as the independent variable (X), working memory as the mediator (M), and age as the moderator (W). Model 59 simultaneously tests the moderating effect of W on the X → M (*a* path), the M → Y (*b* path) and the X → Y direct (*c*) path. A separate model was estimated for each perspective-taking sub-dimension, yielding three moderated mediation analyses in which social problem-solving was the focal outcome. As Maxwell and Cole (2007) have noted, mediation tested with cross-sectional data may misrepresent true causal parameters; the present findings should therefore be interpreted as associational rather than causal.

### 2.2 Participants

Participants were 457 children aged 48–72 months attending preschools and kindergarten classrooms affiliated with the Turkish Ministry of National Education in Bolu, Türkiye. In the Turkish system, these two institutional settings implement different national curricula: preschools (independent early childhood education institutions) follow the 2024 Early Childhood Education Program [Ministry of National Education (MoNE), 2024a], whereas kindergarten classrooms (operating within primary schools) follow the 2024 Türkiye Century Education Model Preschool Education Program [Ministry of National Education (MoNE), 2024b]. Both programs are issued by the Ministry of National Education and target the same developmental period. Participants were recruited through convenience sampling, with schools selected based on accessibility and willingness of administrators to participate. Accordingly, the sample is not statistically representative of the broader population of Turkish preschoolers, and findings should be generalized with appropriate caution. Eligibility required attendance at one of the participating classrooms and written parental consent. Participation was also voluntary at the level of the child. No child was pressed to continue, and any child who declined or wished to stop was free to do so without consequence. No eligible child was excluded from the analyses, and no case was lost through listwise deletion. Of the children, 50.3% were girls (*n* = 230) and 49.7% were boys (*n* = 227). The age distribution was 46.0% in the 48–59-month group (*n* = 210) and 54.0% in the 60–72-month group (*n* = 247). Children aged 48–72 months were eligible to participate; observed ages in the recruited sample ranged from 49 to 71 months (M = 59.86, SD = 4.83). With respect to family income, 34.1% of participants were from low-income families (*n* = 156), 46.8% from middle-income families (*n* = 214) and 19.0% from high-income families (*n* = 87). The full sociodemographic composition of the sample is summarized in Table 1.

**Table 1:** Demographic characteristics of participants (*N* = 457).

Variable	Category	*n* (%)
Gender	Girl	230 (50.3)
	Boy	227 (49.7)
Age group	48–59 months	210 (46.0)
	60–72 months	247 (54.0)
Family income	Low	156 (34.1)
	Middle	214 (46.8)
	High	87 (19.0)
Age (months)	Range/M (SD)	49–71/59.86 (4.83)

*N* = 457. Percentages are based on the total sample.

*A priori* power analysis using the Monte Carlo tool of Schoemann et al. (2017) indicated that detecting a medium-sized indirect effect (*a* = *b* = 0.30) at 0.80 power required at least 162 participants. The present sample of *N* = 457 substantially exceeds this threshold and provides adequate power for moderated mediation tests.

### 2.3 Measures

#### 2.3.1 Perspective-taking test for children

The test developed by Aslan and Köksal Akyol (2016). The test is administered individually to children aged three to five years and comprises three sub-dimensions. The perceptual perspective-taking subscale contains four items, the cognitive perspective-taking subscale contains four items, and the affective perspective-taking subscale contains 16 items grouped into four emotion categories (happiness, sadness, fear, anger), with four items per emotion. The instrument therefore comprises 24 items in total. Each item is scored as correct or incorrect; the maximum score is four for the perceptual and cognitive subscales and 16 for the affective subscale. In the original development study, the KR20 internal-consistency coefficient was 0.71, the test–retest reliability was 0.91, and convergent-validity correlations with the Empathy Scale and the Perspective-Taking Test were 0.80 and 0.73, respectively (Aslan and Köksal Akyol, 2016). In the present sample (*N* = 457), the KR-20 internal-consistency coefficient for the total scale was 0.70.

#### 2.3.2 Self-regulation skills scale: working memory subscale

Working memory was assessed using the working-memory subscale of the Self-Regulation Skills Scale developed by Erol and İvrendi (2018). The full instrument is a 20-item mother-report measure designed for children aged 4–6 years and comprises four subscales: attention (six items), working memory (five items), inhibitory control–emotion (five items) and inhibitory control–behavior (four items). Items are rated on a five-point Likert-type scale (1 = never to 5 = always). Only the five-item working-memory subscale was administered in the present study. In the original development study (*N* = 509), confirmatory factor analysis supported the four-factor structure (*χ*^2^/df = 1.91; RMSEA = 0.07; SRMR = 0.07), and factor loadings for the working-memory items ranged from 0.51 to 0.83. The overall scale demonstrated strong reliability (Cronbach’s *α* = 0.90; test-retest = 0.77), and its concurrent validity coefficient was 0.84 (Erol and İvrendi, 2018). Higher scores on the subscale indicate greater working-memory capacity as perceived by the mother. In the present sample (*N* = 457), Cronbach’s *α* for the working-memory subscale was 0.77. Maternal report was preferred to a direct performance task on two grounds. First, performance tasks measure how efficiently a child holds and manipulates information under standardized testing conditions, whereas caregiver ratings describe how consistently the child uses that capacity across everyday routines, including the peer interactions in which social problem solving occurs (Toplak et al., 2013). Second, mothers rated working memory whereas perspective taking and social problem solving were assessed directly with the children, so the indirect associations reported below cannot be attributed to a single informant.

#### 2.3.3 Wally social problem-solving test

Social problem solving was measured with the Wally Social Problem-Solving Test (Webster-Stratton, 1990), adapted into Turkish by Kayılı and Arı (2015). The test is administered individually and uses pictorial scenarios depicting everyday interpersonal problem situations; parallel forms are available for girls and boys. Children describe verbally how they would respond to each scenario, and responses are scored dichotomously as prosocial or antisocial, yielding a total score. The instrument is grounded in the Interpersonal Cognitive Problem-Solving (ICPS) and Social Information Processing (SIP) frameworks. In the Turkish adaptation (*N* = 699, ages 3–5), KR-20 coefficients were 0.72, 0.79 and 0.81 for three-, four- and five-year-olds, respectively, with content, construct and criterion validity established (Kayılı and Arı, 2015). In the present sample (*N* = 457; 4–6-year-olds), the KR-20 coefficient was 0.72.

### 2.4 Procedure

Data were collected during the 2025–2026 academic year in Bolu, Türkiye. The study was conducted in accordance with the principles of the Declaration of Helsinki. Ethical approval for the study (Protocol No: 2025/676) was granted by the Bolu Abant İzzet Baysal University Ethics Committee for Human Research in the Social Sciences at its meeting dated 29.12.2025 (decision number: 2025/10). Institutional permissions were also obtained prior to data collection. Parents were informed about the study and provided written informed consent for their children’s participation; children also provided verbal assent. The Perspective-Taking Test and the Wally Social Problem-Solving Test were administered individually in school settings, with each session lasting approximately 20–25 min. The Self-Regulation Skills Scale was sent to mothers in sealed envelopes and collected after completion. Children’s attention and motivation were monitored throughout, with brief breaks provided as needed. Sessions were conducted in quiet environments free from distracting stimuli.

### 2.5 Data analysis

Prior to the main analyses, four preliminary checks were conducted to ensure that the data met the assumptions of the planned procedures. First, missing-data patterns were inspected to determine whether listwise deletion or imputation would be required. Second, skewness and kurtosis indices were computed for each continuous variable to evaluate univariate normality, since marked departures from normality can bias parameter estimates and inflate Type I error in regression-based mediation models; values within |skewness| < 2 and |kurtosis| < 7 were treated as supporting the assumption of approximate normality (Kim, 2013). Third, the zero-order correlations among the predictors were examined to detect multicollinearity, which can destabilize regression coefficients in mediation models. Fourth, because formal tests of indirect effects are sensitive to the shape of the sampling distribution, percentile bootstrap confidence intervals based on 5,000 resamples were used for all tests of indirect effects; this procedure does not rely on the assumption of normally distributed sampling distributions (Hayes, 2022) and provides robust inference even under minor distributional irregularities.

Descriptive statistics (means, standard deviations) and Pearson product–moment correlations were computed for all variables. Three separate moderated mediation models were then estimated using PROCESS Model 59 (Hayes, 2022), with each perspective-taking sub-dimension entered in turn as the independent variable, working memory as the mediator, social problem-solving as the outcome and age as the moderator. Age was modeled in two ways. In the primary analyses, age was specified as a dichotomous moderator (48–59 months coded 0; 60–72 months coded 1). This split was determined *a priori* on developmental rather than distributional grounds. The 60-month boundary marks the point by which core Theory of Mind components are typically consolidated (Rakoczy, 2022; Wellman and Liu, 2004) and by which working-memory capacity has undergone its most pronounced expansion (Ahmed et al., 2022; He et al., 2019), so it separates children who differ in the cognitive resources the model concerns. The categorical specification also yields conditional indirect effects for two age bands that correspond to the groupings used in preschool practice, which makes the findings directly interpretable for classroom application. In a second specification, age was entered in months as a continuous moderator. Because dichotomizing a continuous variable reduces statistical power and can distort parameter estimates (MacCallum et al., 2002), all three models were re-estimated in this way and the Johnson–Neyman procedure was applied. The index of moderated mediation tests whether the indirect effect differs significantly across levels of the moderator. All analyses were conducted in IBM SPSS Statistics 26 with PROCESS macro v4.2.

## 3 Results

### 3.1 Descriptive statistics and correlations

The data set was complete on all study variables (*N* = 457), and skewness and kurtosis values for the continuous measures fell within the limits of |skewness| < 2 and |kurtosis| < 7 (Kim, 2013), supporting the assumption of approximate normality. Zero-order correlations among the predictors were moderate but non-redundant (rs = 0.48–0.56), indicating no multicollinearity concerns. Descriptive statistics and Pearson correlations among the study variables are presented in Table 2.

**Table 2:** Descriptive statistics and Pearson correlations.

Variable	*M*	*SD*	1	2	3	4	5
1. Cognitive PT	2.55	1.20	—				
2. Perceptual PT	2.47	1.23	0.53**	—			
3. Affective PT	8.21	4.09	0.56**	0.54**	—		
4. Working Memory	17.53	4.07	0.48**	0.49**	0.48**	—	
5. Social Problem-solving	8.75	3.59	0.33**	0.30**	0.34**	0.43**	—

*N* = 457. PT, perspective taking. Values are raw total scores; possible ranges are 0–4 for cognitive and perceptual perspective taking, 0–16 for affective perspective taking, 5–25 for the working-memory subscale, and 0–15 for social problem solving. Correlations are Pearson product–moment coefficients. ^**^p < 0.01 (two-tailed).

Inspection of Table 2 indicates that all variables included in the study were positively and significantly correlated (*p* < 0.01). Social problem solving was positively associated with cognitive perspective taking (*r* = 0.33), perceptual perspective taking (*r* = 0.30), affective perspective taking (*r* = 0.34) and working memory (*r* = 0.43). The three perspective-taking sub-dimensions were moderately correlated with one another (*r* = 0.53–0.56) and each was positively associated with working memory (*r* = 0.48–0.49).

### 3.2 Cognitive perspective-taking model

As shown in Table 3, cognitive perspective taking significantly predicted working memory (*b* = 1.147, *p* < 0.001), and the X × W1 interaction was significant (*b* = 0.962, *p* = 0.001). In the outcome model, cognitive perspective taking (*b* = 0.435, *p* = 0.024) and working memory (*b* = 0.203, *p* = 0.001) were both positively associated with social problem solving; the M × W1 interaction was significant (*b* = 0.190, *p* = 0.026), whereas X × W1 was not (*b* = 0.022, *p* = 0.938).

**Table 3:** Moderated mediation model for cognitive perspective taking.

Predictor	*b*	SE	*t*	*p*	95% CI [LL, UL]
Panel A. Mediator model (Y = working memory)					
Constant	−0.044	0.244	−0.18	0.858	[−0.523, 0.436]
Cognitive perspective-taking (X)	1.147	0.198	5.81	<0.001	[0.759, 1.536]
Age group (W1)	0.078	0.332	0.24	0.814	[−0.574, 0.730]
X × W1	0.962	0.277	3.47	0.001	[0.418, 1.505]
Model summary: *R* = 0.502, *R*^2^ = 0.252, MSE = 12.496, *F*(3, 453) = 50.761, *p* < 0.001. Δ*R*^2^ for the X × W1 interaction = 0.020, *F*(1, 453) = 12.074, *p* = 0.001.					
Panel B. Outcome model (Y = social problem solving)					
Constant	8.964	0.220	40.77	<0.001	[8.532, 9.396]
Cognitive perspective-taking (X)	0.435	0.192	2.26	0.024	[0.057, 0.812]
Working memory (M)	0.203	0.063	3.22	0.001	[0.079, 0.327]
Age group (W1)	−0.402	0.299	−1.35	0.179	[−0.990, 0.185]
X × W1	0.022	0.286	0.08	0.938	[−0.541, 0.585]
M × W1	0.190	0.085	2.23	0.026	[0.023, 0.357]

Model summary: *R* = 0.469, *R*^2^ = 0.220, MSE = 10.150, *F*(5, 451) = 25.412, *p* < 0.001. Δ*R*^2^ for the interactions: X × W1 = 0.000, *F*(1, 451) = 0.006, *p* = 0.938; M × W1 = 0.009, *F*(1, 451) = 4.978, *p* = 0.026.

X = cognitive PT; M = working memory; Y = social problem solving; W1 = age group (0 = 48–59 months, 1 = 60–72 months). Unstandardized coefficients; 5,000 bootstrap resamples (Hayes, 2022). CI = confidence interval. The conditional effects derived from this model are reported in Table 4.

As shown in Table 4, the conditional a path was *b* = 1.147 and *b* = 2.109 (both *p* < 0.001) and the conditional *b* path was *b* = 0.203 (*p* = 0.001) and *b* = 0.393 (*p* < 0.001) in the younger and older groups, respectively. Conditional direct effects (*c*′) were significant in both groups (*b* = 0.435, *p* = 0.024; *b* = 0.457, *p* = 0.032). The conditional indirect effect was 0.233, 95% CI [0.081, 0.409] in the younger and 0.828, 95% CI [0.577, 1.112] in the older group; the index of moderated mediation was 0.595, 95% CI [0.292, 0.925].

**Table 4:** Conditional effects for the cognitive perspective-taking model.

Panel A. Conditional *a*-path: cognitive PT → working memory					
Age group	Effect (*b*)	*SE*	*t*	*p*	95% CI [LL, UL]
48–59 months	1.147	0.198	5.79	<0.001	[0.759, 1.535]
60–72 months	2.109	0.194	10.87	<0.001	[1.728, 2.489]

Panel B. Conditional *b*-path: working memory → SPS					
Age group	Effect (*b*)	SE	*t*	*p*	95% CI [LL, UL]
48–59 months	0.203	0.063	3.22	0.001	[0.079, 0.327]
60–72 months	0.393	0.057	6.85	<0.001	[0.280, 0.505]

Panel C. Conditional direct effect (*c*′): cognitive PT → SPS					
Age group	Effect (*b*)	SE	*t*	*p*	95% CI [LL, UL]
48–59 months	0.435	0.192	2.26	0.024	[0.057, 0.812]
60–72 months	0.457	0.212	2.15	0.032	[0.040, 0.874]

Panel D. Conditional indirect effect (*a* × *b*): cognitive perspective taking → working memory → social problem solving			
Age group	Indirect effect	BootSE	95% CI [LL, UL]
48–59 months	0.233	0.084	[0.081, 0.409]
60–72 months	0.828	0.136	[0.577, 1.112]

Panel E. Index of moderated mediation (difference in indirect effects)			
Age group	Value	BootSE	95% CI [LL, UL]
W1	0.595	0.161	[0.292, 0.925]

Conditional *a*-path, *b*-path, direct (*c*′) and indirect (*a* × *b*) effects at each level of the moderator, derived from the model summarized in Table 3. 95% bootstrap confidence intervals (5,000 resamples).

### 3.3 Perceptual perspective-taking model

As shown in Table 5, perceptual perspective taking significantly predicted working memory (*b* = 1.199, *p* < 0.001), with a significant X × W1 interaction (*b* = 0.783, *p* = 0.004). In the outcome model, perceptual perspective taking did not significantly predict social problem-solving (*b* = 0.214, *p* = 0.283); working memory significantly predicted social problem-solving (*b* = 0.230, *p* < 0.001); M × W1 was significant (*b* = 0.173, *p* = 0.044); X × W1 was not (*b* = 0.143, *p* = 0.614).

**Table 5:** Moderated mediation model for perceptual perspective taking.

Predictor	*b*	SE	*t*	*p*	95% CI [LL, UL]
Panel A. Mediator model (Y = working memory)					
Constant	−0.112	0.243	−0.46	0.645	[−0.590, 0.366]
Perceptual perspective-taking (X)	1.199	0.203	5.92	<0.001	[0.801, 1.597]
Age group (W1)	0.243	0.331	0.73	0.463	[−0.407, 0.893]
X × W1	0.783	0.271	2.89	0.004	[0.251, 1.315]
Model summary: *R* = 0.507, *R*^2^ = 0.257, MSE = 12.404, *F*(3, 453) = 52.266, *p* < 0.001. Δ*R*^2^ for the X × W1 interaction = 0.014, *F*(1, 453) = 8.354, *p* = 0.004.					
Panel B. Outcome model (Y = social problem-solving)					
Constant	8.952	0.222	40.42	<0.001	[8.517, 9.387]
Perceptual perspective taking (X)	0.214	0.199	1.08	0.283	[−0.178, 0.606]
Working memory (M)	0.230	0.063	3.63	<0.001	[0.105, 0.355]
Age group (W1)	−0.373	0.301	−1.24	0.216	[−0.965, 0.219]
X × W1	0.143	0.282	0.50	0.614	[−0.412, 0.697]
M × W1	0.173	0.086	2.02	0.044	[0.004, 0.342]

Model summary: *R* = 0.459, *R*^2^ = 0.211, MSE = 10.271, *F*(5, 451) = 24.059, *p* < 0.001. Δ*R*^2^ for the interactions: X × W1 = 0.000, *F*(1, 451) = 0.255, *p* = 0.614; M × W1 = 0.007, *F*(1, 451) = 4.063, *p* = 0.044.

X = perceptual PT; M = working memory; Y = social problem solving; W1 = age group (0 = 48–59 months, 1 = 60–72 months). Unstandardized coefficients; 5,000 bootstrap resamples. CI = confidence interval. The conditional effects derived from this model are reported in Table 6.

As shown in Table 6, the conditional a path was *b* = 1.199 and *b* = 1.982 (both *p* < 0.001) and the conditional *b* path was *b* = 0.230 and *b* = 0.403 (both *p* < 0.001) in the younger and older groups. The conditional direct effect was non-significant in both groups (*b* = 0.214, *p* = 0.283; *b* = 0.357, *p* = 0.075). The conditional indirect effect was 0.276, 95% CI [0.115, 0.469] in the younger and 0.799, 95% CI [0.546, 1.063] in the older group; the index of moderated mediation was 0.523, 95% CI [0.211, 0.839].

**Table 6:** Conditional effects for the perceptual perspective-taking model.

Panel A. Conditional *a*-path: perceptual PT → working memory					
Age group	Effect (*b*)	SE	*t*	*p*	95% CI [LL, UL]
48–59 months	1.199	0.203	5.91	<0.001	[0.801, 1.597]
60–72 months	1.982	0.180	11.01	<0.001	[1.629, 2.335]

Panel B. Conditional *b*-path: working memory → SPS					
Age group	Effect (*b*)	*SE*	*t*	*p*	95% CI [LL, UL]
48–59 months	0.230	0.063	3.63	<0.001	[0.105, 0.355]
60–72 months	0.403	0.058	6.95	<0.001	[0.289, 0.517]

Panel C. Conditional direct effect (*c*′): perceptual PT → SPS					
Age group	Effect (*b*)	*SE*	*t*	*p*	95% CI [LL, UL]
48–59 months	0.214	0.199	1.08	0.283	[−0.178, 0.606]
60–72 months	0.357	0.200	1.79	0.075	[−0.036, 0.749]

Panel D. Conditional indirect effect (*a* × *b*): perceptual perspective taking → working memory → social problem solving			
Age group	Indirect effect	BootSE	95% CI [LL, UL]
48–59 months	0.276	0.090	[0.115, 0.469]
60–72 months	0.799	0.132	[0.546, 1.063]

Panel E. Index of moderated mediation (difference in indirect effects)			
Age group	Value	BootSE	95% CI [LL, UL]
W1	0.523	0.161	[0.211, 0.839]

Conditional effects derived from the model summarized in Table 5. 95% bootstrap confidence intervals (5,000 resamples).

### 3.4 Affective perspective-taking model

As shown in Table 7, affective perspective taking significantly predicted working memory (*b* = 0.361, *p* < 0.001), with a significant X × W1 interaction (*b* = 0.228, *p* = 0.005). In the outcome model, affective perspective taking (*b* = 0.127, *p* = 0.027) and working memory (*b* = 0.200, *p* = 0.002) were both positively associated with social problem solving; M × W1 was significant (*b* = 0.177, *p* = 0.038); X × W1 was not (*b* = 0.043, *p* = 0.607).

**Table 7:** Moderated mediation model for affective perspective taking.

Predictor	*b*	*SE*	*t*	*p*	95% CI [LL, UL]
Panel A. Mediator model (Y = working memory)					
Constant	0.024	0.245	0.10	0.922	[−0.458, 0.506]
Affective perspective-taking (X)	0.361	0.059	6.16	<0.001	[0.246, 0.476]
Age group (W1)	−0.083	0.334	−0.25	0.803	[−0.739, 0.572]
X × W1	0.228	0.081	2.80	0.005	[0.068, 0.388]
Model summary: *R* = 0.495, *R*^2^ = 0.245, MSE = 12.611, *F*(3, 453) = 48.932, *p* < 0.001. Δ*R*^2^ for the X × W1 interaction = 0.013, *F*(1, 453) = 7.845, *p* = 0.005.					
Panel B. Outcome model (Y = social problem-solving)					
Constant	8.988	0.219	40.97	<0.001	[8.556, 9.419]
Affective perspective-taking (X)	0.127	0.057	2.23	0.027	[0.015, 0.240]
Working memory (M)	0.200	0.063	3.15	0.002	[0.075, 0.324]
Age group (W1)	−0.453	0.298	−1.52	0.130	[−1.039, 0.134]
X × W1	0.043	0.083	0.51	0.607	[−0.121, 0.206]
M × W1	0.177	0.085	2.08	0.038	[0.010, 0.343]

Model summary: *R* = 0.474, *R*^2^ = 0.225, MSE = 10.082, *F*(5, 451) = 26.202, *p* < 0.001. Δ*R*^2^ for the interactions: X × W1 = 0.001, *F*(1, 451) = 0.265, *p* = 0.607; M × W1 = 0.008, *F*(1, 451) = 4.346, *p* = 0.038.

X = Affective PT; M = Working memory; Y = Social problem solving; W1 = Age group (0 = 48–59 months, 1 = 60–72 months). Unstandardized coefficients; 5,000 bootstrap resamples. CI, confidence interval. The conditional effects derived from this model are reported in Table 8.

As shown in Table 8, the conditional a path was *b* = 0.361 and *b* = 0.589 (both *p* < 0.001) and the conditional *b* path was *b* = 0.200 (*p* = 0.002) and *b* = 0.376 (*p* < 0.001) in the younger and older groups. The conditional direct effect was significant in both groups (*b* = 0.127, *p* = 0.027; *b* = 0.170, *p* = 0.005). The conditional indirect effect was 0.072, 95% CI [0.026, 0.126] in the younger and 0.222, 95% CI [0.148, 0.307] in the older group; the index of moderated mediation was 0.150, 95% CI [0.057, 0.246].

**Table 8:** Conditional effects for the affective perspective-taking model.

Panel A. Conditional *a*-path: affective PT → working memory					
Age group	Effect (*b*)	SE	*t*	*p*	95% CI [LL, UL]
48–59 months	0.361	0.059	6.16	<0.001	[0.246, 0.476]
60–72 months	0.589	0.057	10.43	<0.001	[0.478, 0.700]

Panel B. Conditional *b*-path: working memory → SPS					
Age group	Effect (*b*)	SE	*t*	*p*	95% CI [LL, UL]
48–59 months	0.200	0.063	3.15	0.002	[0.075, 0.324]
60–72 months	0.376	0.056	6.71	<0.001	[0.266, 0.486]

Panel C. Conditional direct effect (*c*′): affective PT → SPS					
Age group	Effect (*b*)	SE	*t*	*p*	95% CI [LL, UL]
48–59 months	0.127	0.057	2.23	0.027	[0.015, 0.240]
60–72 months	0.170	0.060	2.82	0.005	[0.051, 0.289]

Panel D. Conditional indirect effect (*a* × *b*): affective perspective taking → working memory → social problem solving			
Age group	Indirect effect	BootSE	95% CI [LL, UL]
48–59 months	0.072	0.026	[0.026, 0.126]
60–72 months	0.222	0.041	[0.148, 0.307]

Panel E. Index of moderated mediation (difference in indirect effects)			
Age group	Value	BootSE	95% CI [LL, UL]
W1	0.150	0.049	[0.057, 0.246]

Conditional effects derived from the model summarized in Table 7. 95% bootstrap confidence intervals (5,000 resamples).

### 3.5 Continuous age moderator (Johnson–Neyman)

All three moderated mediation models were re-estimated with age entered as a continuous moderator (W = age in months) and the Johnson–Neyman procedure was applied; full results are presented in Table 9. The first-stage (*a*-path) interaction was significant in all three models. The Johnson–Neyman transition fell at 49.64 months in the cognitive model, and no transition point was located within the observed age range in the perceptual and affective models, in which the conditional effect was significant throughout. The second-stage (*b*-path) interaction did not reach the conventional threshold in any model (cognitive *b* = 0.014, *p* = 0.121; perceptual *b* = 0.016, *p* = 0.079; affective *b* = 0.015, *p* = 0.074). The Johnson–Neyman procedure nevertheless identified the age at which the conditional *b*-path effect became significant, at 50.61 months in the perceptual model and 51.01 months in the affective model, whereas no such region was defined in the cognitive model. Conditional indirect effects at the 16th, 50th and 84th age percentiles were positive in every model, and pairwise bootstrap contrasts confirmed that the indirect effect at 65 months exceeded that at 54 months across all three sub-dimensions.

**Table 9:** Continuous-age moderator with Johnson–Neyman probing.

Measure	Cognitive PT	Perceptual PT	Affective PT
X × W (age in months), *b* (SE)	0.101 (0.028)	0.083 (0.028)	0.017 (0.008)
*p* value (X × W)	<0.001	0.003	0.039
M × W (working memory × age in months), *b* (SE)	0.014 (0.009)	0.016 (0.009)	0.015 (0.008)
*p* value (M × W)	0.121	0.079	0.074
J–N transition, *a*-path (months; % above)	49.64 (99.1%)	None (100%)	None (100%)
J–N transition, *b*-path (months; % above)	n/a (*p* ≥ 0.10)	50.61 (97.6%)	51.01 (95.4%)
Indirect effect @ 54 mo [95% BootCI]	0.247 [0.100, 0.425]	0.278 [0.112, 0.470]	0.082 [0.029, 0.148]
Indirect effect @ 60 mo [95% BootCI]	0.528 [0.376, 0.696]	0.558 [0.404, 0.726]	0.148 [0.103, 0.198]
Indirect effect @ 65 mo [95% BootCI]	0.839 [0.552, 1.163]	0.866 [0.583, 1.163]	0.217 [0.141, 0.305]

PROCESS Model 59 (Hayes, 2022); W = age in months. *a*-path = X → M; *b*-path = M → Y. Conditional indirect effects are reported at the 16th, 50th and 84th age percentiles with 95% bootstrap CIs (5,000 resamples). Johnson–Neyman transition = age at which the conditional path first reaches significance at *α* = 0.05; percentages refer to the proportion of the sample at or above this age. “none” = path significant across the full age range; “n/a” = the interaction did not meet the PROCESS probing threshold of *p* < 0.10, so no transition point was computed.

### 3.6 Robustness checks

The primary findings were supported by two robustness procedures that did not require re-analysis of the raw data. First, all three indices of moderated mediation were supported by bootstrap 95% confidence intervals that excluded zero. Second, the pattern of conditional indirect effects was consistent across the three perspective-taking sub-dimensions, indicating that the moderated-mediation structure was not driven by a single measure.

## 4 Discussion

The present study examined the indirect role of working memory in the links between three sub-dimensions of perspective taking and social problem solving in early childhood, and tested whether this pathway is moderated by age. The findings reveal a consistent pattern across the three models. The association between perspective taking and social problem solving was in part indirect, running through working memory, and the strength of this indirect association varied with age (Hayes, 2022). The Discussion is organized to mirror the structure of the Results. The bivariate pattern is considered first, then each of the three models in turn, followed by robustness, an integrative interpretation, implications, and limitations.

### 4.1 Descriptive pattern and replication of established associations

At the bivariate level, the three perspective-taking sub-dimensions were positively related to working memory and to social problem solving, and working memory was positively related to social problem solving. The magnitudes of these zero-order correlations were moderate and internally coherent, consistent with the view that these constructs form a developmentally interdependent network during the preschool years. The pattern accords with He et al.’s (2019) demonstration that working memory shows a marked increase between four and five years, and with Grosse Wiesmann et al.’s (2020) neuroimaging evidence that structural maturation of temporoparietal and precuneal cortex supports the emerging capacity to represent others’ mental states. The moderate rather than high size of the correlations is theoretically informative. It indicates shared variance without conflation, arguing against reducing the three constructs to a single underlying factor and motivating the moderated-mediation analyses that follow. Together, these bivariate associations replicate the established perspective-taking-SPS and working memory-SPS links documented in predominantly Western samples (H1 and H2 supported), confirming that the foundational relationships generalize to a non-Western preschool population.

### 4.2 Cognitive perspective-taking model

The cognitive perspective-taking model produced the largest index of moderated mediation. Cognitive perspective taking significantly predicted working memory, the association was stronger in older preschoolers, and the indirect pathway through working memory was substantial and conditional on age. In parallel, the direct effect on social problem solving was preserved in both age groups. Cognitive perspective taking is therefore linked with social problem solving through both direct and working-memory-mediated indirect channels simultaneously, and the indirect channel gains weight across the preschool years. This pattern is theoretically expected. Cognitive perspective taking inherently requires the construction and maintenance of mental representations of others’ beliefs and intentions, so it benefits most from developmental gains in working memory (Rakoczy, 2022). Ertuğrul-Yaşar's (2024) finding that distinct components of ToM follow differentiated developmental trajectories and Nejati et al.’s (2024) evidence that the relation between executive function and ToM varies by age and cultural context further support this interpretation.

### 4.3 Perceptual perspective-taking model

In the perceptual model, the direct effect of perspective taking on social problem solving was non-significant in both age groups, while the indirect effect through working memory was significant and strengthened with age. According to the Zhao et al. (2010) classification, this pattern corresponds to “indirect-only” mediation, suggesting that the association between perceptual perspective taking and social problem solving is almost entirely indirect, running through working memory. Rucker et al. (2011) caution that contemporary mediation frameworks treat the full-partial distinction as less critical than the magnitude and theoretical meaning of the indirect effect. Perceptual perspective taking is a more elementary cognitive ability concerned with adopting another’s physical vantage point (Flavell, 2004; Healey and Grossman, 2018; Li et al., 2024). Its association with a complex process such as social problem solving is therefore expected to depend more heavily on working-memory-mediated indirect routes. He et al. (2024) demonstrated experimentally that verbal and spatial working memory play a critical role in perceptual perspective taking and that this relation varies with age, paralleling the age-sensitive indirect effect observed here.

### 4.4 Affective perspective-taking model

The affective perspective-taking model produced the smallest index of moderated mediation, though the pattern remained consistent with a working-memory-mediated indirect pathway that strengthens with age. The direct effect was preserved in both age groups, indicating that affective perspective taking links to social problem solving through both direct and indirect channels simultaneously. The comparatively smaller mediated contribution may reflect the earlier developmental maturation of emotion recognition and emotion sharing (Decety and Holvoet, 2021; Lucas-Molina et al., 2024) and a correspondingly weaker dependence of affective perspective taking on developmental changes in working-memory capacity. Even so, the stronger indirect effect in the older group shows that the general developmental trend is preserved within this sub-dimension as well. The asymmetry across the three models is theoretically informative. Affective perspective taking emerges early and is comparatively automatic, with limited reliance on prefrontal executive control (Decety and Holvoet, 2021; Healey and Grossman, 2018), and by 48–72 months it is already partially consolidated. Cognitive perspective taking, by contrast, consolidates only around four to five years (Rakoczy, 2022; Wellman, 2018) and remains contingent on executive support thereafter (Devine and Hughes, 2014; Pesch et al., 2020). Perceptual perspective taking occupies an intermediate position, with working memory shown to support level-1 visual perspective-taking tasks in children (He et al., 2024). The three sub-dimensions are therefore not functionally interchangeable inputs into social problem solving, supporting the analytic decision to model them separately.

### 4.5 Robustness and alternative explanations

Across the three models, H3 (the perspective-taking → social problem solving association is indirect, operating through working memory), H4 (age moderates the a-path), and H6 (significant index of moderated mediation) were supported for all three perspective-taking sub-dimensions. H5 (age moderates the *b*-path) was supported in the categorical age-group model (cognitive *b* = 0.190, *p* = 0.026; perceptual *b* = 0.173, *p* = 0.044; affective *b* = 0.177, *p* = 0.038), but the corresponding interaction did not reach conventional significance in the continuous-age (Johnson–Neyman) specification, indicating partial support. Bootstrap 95% confidence intervals for all three indices of moderated mediation excluded zero, and the pattern of conditional indirect effects was consistent across the three sub-dimensions, indicating that the moderated-mediation structure was not driven by a single measure. The conditional indirect effects showed the same age-graded pattern when age was re-entered as a continuous moderator and the Johnson–Neyman procedure was applied. The conditional indirect effect was positive and significant at each age probed (54, 60 and 65 months) and increased in magnitude across that range. The conditional *b*-path effect, by contrast, reached significance at approximately 50–51 months in the perceptual and affective models. This transition window coincides with the documented expansion of working memory between four and five years (He et al., 2019), with structural maturation of temporoparietal and precuneal cortex supporting the emerging capacity to represent others’ mental states (Grosse Wiesmann et al., 2020), and with evidence that executive functioning undergoes qualitative reorganization in the preschool years (Devine and Hughes, 2014). The moderate correlations among the three constructs invite the concern that a general cognitive ability factor might account for the observed associations. Three observations weaken this account. First, the indirect-effect coefficients differed across the three sub-dimensions, whereas a single general factor would predict a homogeneous pattern. Second, the absence of a direct effect in the perceptual model alongside its preservation in the cognitive and affective models is inconsistent with a single-factor account. Third, the path-specific operation of age moderation (*a*-path versus *b*-path) points to sub-process-specific developmental mechanisms beyond a unitary ability factor. Together, these observations support a sub-dimension-specific reading of the findings rather than a reductive executive-function account.

### 4.6 Integrative interpretation

A notable feature of the present findings concerns the direct pathway from perspective taking to social problem solving. The age-moderation of this pathway was tested in an exploratory manner (H7), and the direct effect did not weaken with age in any of the three models. The indirect route through working memory is evident in both age groups and is substantially stronger in the older one. The developmental shift is therefore additive rather than substitutive. The indirect pathway is added to, rather than replacing, the existing direct pathway. De Lillo and Ferguson (2023) reported a similar pattern in which executive functions contribute to perspective-taking development without entirely supplanting the direct pathway, and Morín and Keulers (2024) likewise identified executive function and working memory as key predictors of ToM development. Older children develop more complex, multi-step strategies (Ayık et al., 2025; Rubin and Rose-Krasnor, 1992), and this qualitative differentiation is closely tied to the expansion of working-memory capacity, since multi-step strategies require multiple sources of information to be processed simultaneously (Dodge, 1986; Schäfer et al., 2024). Theoretically, this pattern aligns with positions that view working memory as an integrative rather than purely storage system in social cognition (Baddeley, 2003; Doebel, 2020). The pattern also coheres with the developmental cascades framework (Masten and Cicchetti, 2010), in which foundational capacities scaffold the emergence of more complex systems without erasing their predecessors.

### 4.7 Theoretical and applied implications

Theoretically, the findings support the view that social problem solving is better understood as a complex outcome of interrelated cognitive mechanisms than as the product of a single ability (D'Zurilla and Goldfried, 1971; Dodge, 1986; Doebel, 2020; Rubin and Krasnor, 1986). The complementary roles of perspective taking and working memory underscore the need for integrative frameworks in models of social cognition (Imuta et al., 2016). The moderating effect of age also indicates that age should be modeled as a contextual variable that conditions these associations rather than as a simple control (Devine and Hughes, 2014; Hayes, 2022).

Practically, the pattern observed here is consistent with intervention approaches that combine perspective-taking activities with play-based working-memory tasks (Walk et al., 2018), and perspective-taking instruction has been shown to enhance social cohesion (Alan et al., 2021). Because the present design is cross-sectional, these implications are provisional and indicate directions for intervention research rather than established curricular prescriptions. If such approaches are pursued, they could be developmentally graded. Activities for younger children could focus on direct mappings between perspective taking and social problem situations, whereas programs for older children could combine perspective-taking activities with working-memory-strengthening tasks. The Turkish cultural context adds an important layer to this practical translation. Urban Turkish socialization synthesizes autonomy and relatedness orientations (Hofstede et al., 2010; Kağıtçıbaşı, 2017), and Turkish preschool classrooms have historically prioritized motor and cognitive outcomes over socioemotional competence, with teachers seldom encouraging discussion or problem-solving during peer conflicts (Bugan-Kisir and Corapci, 2026). Activities of this kind, embedded in classroom routines, may therefore address a structural gap rather than simply add new content. At the policy level, the OECD’s Starting Strong V framework (OECD, 2017) and the UNESCO Education 2030 agenda (UNESCO, 2016) emphasize the integration of social–emotional and cognitive foundations in early childhood interventions. The present findings contribute to that discussion by identifying working memory as a candidate for closer attention. The Türkiye Yüzyılı Maarif Modeli [Ministry of National Education (MoNE), 2024b] defines social–emotional development as a learning domain and highlights self-regulation, yet does not explicitly operationalize executive-function-based activities within the early-childhood component. Whether addressing this gap would improve social problem solving in children is an empirical question that requires experimental evaluation. Korucu et al. (2017) reported strong links between self-regulation and social-cognitive skills in Turkish samples, and the present findings are consistent with that pattern. At the assessment level, including working-memory and perspective-taking measures in the evaluation of children’s social problem-solving abilities supports a more comprehensive profile (Aslan and Köksal Akyol, 2016; Erol and İvrendi, 2018) and may inform the targeting of early-intervention programs (Ayık et al., 2025; Gülay Ogelman et al., 2023; Kağıtçıbaşı, 2017).

### 4.8 Limitations and future directions

Several limitations should be acknowledged. First, the cross-sectional design precludes causal inference. Because perspective taking, working memory and social problem solving were measured concurrently, the temporal-precedence assumption underlying mediation cannot be empirically tested (Maxwell and Cole, 2007). Second, working memory was assessed by maternal report. Ratings of executive function correspond only modestly to performance-based tasks, with a median correlation of 0.19 across 20 studies (Toplak et al., 2013), and maternal ratings may be shaped by parental expectations about the child. The present estimates therefore describe the everyday expression of working memory rather than its capacity under standardized testing conditions. Third, the sample was drawn from a single province in northwestern Türkiye through convenience sampling. The resulting sample does not capture the socioeconomic and regional diversity of Turkish preschoolers, so the findings should not be generalized to the national population. Fourth, the same researcher administered and scored the child measures and no independent second coder was employed for the Wally responses, so inter-rater reliability and formal blinding could not be documented, and expectancy effects on scoring cannot be ruled out. Fifth, formal measurement invariance across gender and age was not tested, so the observed age differences may partly reflect differences in how the instruments function across age groups rather than differences in the constructs themselves. Sixth, the executive-function system was represented only by working memory. Inhibitory control and cognitive flexibility were not modeled (Schäfer et al., 2024). Future research should address these limitations through longitudinal designs that treat age as a continuous variable, through the inclusion of inhibitory control and cognitive flexibility, through direct performance measures of working memory, and through independent double scoring of the child measures. Testing similar models in different socioeconomic and cultural contexts will provide stronger evidence regarding generalizability, and intervention research using longitudinal designs to test the effect of working-memory training on social problem-solving development will allow the present indirect associations to be examined experimentally.

## 5 Conclusion

This study examined the indirect role of working memory in the relation between perspective-taking sub-dimensions and social problem solving in early childhood, together with the moderating role of age. The findings show that the associations of all three perspective-taking sub-dimensions with social problem solving were in part indirect, running through working memory, and that this indirect association was stronger in 60- to 72-month-old children. The direct pathway, by contrast, remained stable across age, indicating an additive rather than substitutive developmental shift. Across all three models, bootstrap 95% confidence intervals for the indices of moderated mediation excluded zero, indicating that the strength of the indirect association differed by age group. By modeling the three perspective-taking sub-dimensions, working memory and social problem solving together, and by allowing age to condition that model, the study specifies where in the preschool period the indirect association is most pronounced. Its cross-sectional design leaves the developmental ordering of these variables open to longitudinal test.

## Data Availability

The anonymized dataset analyzed in this study is available from the corresponding author on reasonable request. As the data were collected from preschool-aged children, requests are reviewed by the corresponding author to ensure that participant confidentiality is preserved. Requests to access the dataset should be directed to mfatihkucukkara@ef.ibu.edu.tr.
